# A four prognosis-associated lncRNAs (PALnc) based risk score system reflects immune cell infiltration and predicts patient survival in pancreatic cancer

**DOI:** 10.1186/s12935-020-01588-y

**Published:** 2020-10-09

**Authors:** Hongkai Zhuang, Shanzhou Huang, Zixuan Zhou, Zuyi Ma, Zedan Zhang, Chuanzhao Zhang, Baohua Hou

**Affiliations:** 1 Department of General Surgery, Guangdong Provincial People’s Hospital, Guangdong Academy of Medical Sciences, No. 106 Zhongshan Er Road, Guangzhou, 510080 China; 2 Shantou University of Medical College, Shantou, 515000 Guangdong China; 3 South China University of Technology School of Medicine, Guangzhou, 510006 Guangdong China

**Keywords:** Pancreatic cancer, Biomarker, lncRNAs, Risk score, TCGA

## Abstract

**Background:**

Pancreatic cancer (PC) is one of the most common cancers and the leading cause of cancer-related death worldwide. Exploring novel predictive biomarkers for PC patients’ prognosis is in urgent need.

**Methods:**

In the present study, we conducted Cox proportional hazards regression to identify critical prognosis-associated lncRNAs (PALncs) in TCGA PC dataset. Based on the results of multivariate analysis, a PALnc-based risk score system was established, and validated in GSE62452 dataset. The validity and reliability of the risk score system for prognosis of PC were evaluated through ROC analysis. And function enrichment analyses for the PALncs were also performed.

**Result:**

In the multivariate analysis, four PALncs (LINC00476, C9orf163, LINC00346 and DSCR9) were screened out to develop a risk score system, which showed a high AUC at 3 and 5 years overall survival (0.785 at 3 year OS, 0.863 at 5 year OS) in TCGA datasets. And the ROC analysis of the risk score system for RFS in TCGA dataset revealed that AUC for RFS was 0.799 at 3 years and 0.909 at 5 years. Further, the AUC for OS in the validation cohort was 0.705 at 3 years and 0.959 at 5 years. Furthermore, the functional enrichment analysis revealed that these PALncs may be involved in various pathways related to cancer, including Ras family activation, autophagy in cancer, MAPK signaling pathway, HIF-1 signaling pathway, PI3K-Akt signaling pathway, etc. And correlation analysis of these tumor infiltrating immune cells and risk score system revealed that the infiltration level of B cell naïve, plasma cells, and CD8+ T cells are negatively correlated to the risk score system, while macrophages M2 positively correlated to the risk score system.

**Conclusion:**

Our study established a four PALncs based risk score system, which reflects immune cell infiltration and predicts patient survival for PC.

## Background

As one of the most aggressive cancers, pancreatic cancer (PC) caused 4.5% of all cancer related deaths in 2015 globally [1]. The prognosis of PC patients could be varied due to the characteristic of heterogeneity. There are some clinical risk factors, e.g. tumor size, vessel invasion, etc., which are associated with patients survival [2]. However, these clinical factors do not offer information on cancer cell’s biological behavior, leading to less precise predictive value for oncology outcome. Thus, it is an urgent need to find out novel biomarkers and develop more precise models for predicting patients’ prognosis for PC [3].

Long non-coding RNAs (lncRNAs) are a type of RNA with lengths exceeding 200 nucleotides, most of which are not translated into protein. LncRNAs are involved in regulating gene expression at either transcriptional or post-transcriptional level [4]. Studies have found that some lncRNAs play a role in PC initiation and progression. For example, LncRNA-BX111 promotes metastasis of pancreatic cancer through regulating epithelia-mesenchymal transition (EMT) [5]. LncRNA UCA1 was reported to promote migration and invasion in pancreatic cancer cells via the Hippo pathway [6]. However, the clinical relevance as well as predictive value of LncRNAs in PC is not well demonstrated.

By performing multiple bioinformatics analysis in the study, 4 prognosis-associated LncRNAs (PALncs) were identified from TCGA PC datasets to establish a 4-PALncs based risk score system for prediction of prognosis for PC. The predictive power of this risk score system was assessed in TCGA dataset and was validated in GEO dataset. In order to gain insight into the carcinogenesis of the PALncs in PC, we also explored the molecular mechanisms related to the correlated genes of the four PALncs by GO enrichment analysis and pathway analysis.

## Materials and methods

### Data source

The mRNA sequencing data (178 PC samples and 4 pancreatic normal tissues) of pancreatic cancer (PC) (platform: Illumina Hiseq 2000 RNA Sequencing; downloaded on May 3, 2019) and correlated clinical information were obtained from The Cancer Genome Atlas (TCGA, https://cancergenome.nih.gov/) database, which was set as the training dataset. Relevant PC datasets were also extracted from the Gene Expression Omnibus (GEO, https://www.ncbi.nlm.nih.gov/geo/) database by using the following search words: “pancreatic cancer” and “pancreatic ductal adenocarcinoma.” The exclusion of ineligible studies was based on the following criteria: (1) studies with fewer than 30 PC samples and non-tumor pancreatic samples; (2) studies involving only blood samples or tumor samples; (3) studies using only PC cell lines or xenografts; and (4) no clinical information. Finally, GSE62452 [platform: GPL6244 (HuGene-1_0-st) Affymetrix Human Gene 1.0 ST Array [transcript (gene) version]; 61 non-tumor samples and 69 tumor samples] and GSE60979 (platform: GPL14550 SurePrint G3 Human GE 8 × 60 K Microarray; 12 non-tumor samples and 49 pancreatic cancer samples) were selected for further study. Probe IDs were matched with the gene symbols consisting of the annotation file provided by the manufacturer. If multiple probe IDs matched a single gene, probe IDs were merged using the arithmetic mean of the expression level of a single gene. The two GEO datasets were integrated into meta-GEO PC dataset (73 non-tumor samples and 118 tumor samples). And the sva package (Version: 3.30.1; https://bioconductor.org/packages/release/bioc/html/sva.html) was used to eliminate batch effects. The scale method of the R package limma (Version 3.38.3; https://www.bioconductor.org/packages/release/bioc/html/limma.html) was used to normalize the data. All datasets (TCGA, GSE62452, and GSE60979) are freely available as public resources. Therefore, local ethics approval was not needed.

### Differential expression analysis

After alignment to the human genome (Ensemble genome browser 90), we obtained 14,435 lncRNAs based on their Transcript stable ID and Gene stable ID in TCGA PC dataset. Then, we screened out 186 lncRNAs from meta-GEO PC dataset through matching the gene symbols of meta-GEO PC dataset with the human genome (Ensemble genome browser 90). And the R package limma (Version 3.38.3; https://www.bioconductor.org/packages/release/bioc/html/limma.html) was used to find out the differentially expressed lncRNAs (DELnc) in meta-GEO PC dataset between PC tissues and normal tissues under the thresholds of p-value < 0.05.

### Identification of prognosis-associated lncRNAs (PALnc)

Among 178 PC samples, 177 PC sample had correlated clinical information. And we removed the cases with survival time less than 30 days to avoid the possible influence of surgical complications. Finally we got 172 PC cases with overall survival information. And among them, 145 cases were offered with information of recurrence-free survival (RFS). The univariable Cox proportional regression was performed to evaluate the association between individual DELnc expression and patients’ overall survival (OS) in batches using the R packages survival, survminer, and parallel in R version 3.5.2 (https://www.r-project.org/). A p-value < 0.01 was considered as statistically significant association.

### Establishment and validation of the PALnc-based risk score system

With the help of the R packages survival in R version 3.5.2 (https://www.r-project.org/), a multivariate Cox proportional regression model was established through cycle computation, which had highest discriminated ability for OS of PC. The PALnc-based risk score system was established based on a linear combination of the expression values of the PALncs and the multivariable Cox regression coefficients were used as the weight. The Kaplan–Meier survival curve was performed to validate the correlation between OS and the selected PALncs according to the optimal cutoff point obtained from X-tile [7]. For the external validation cohort for the risk score system, the GSE62452 dataset was analyzed. The risk scores of the PC patients in the validation datasets were assessed using the coefficients acquired from the training dataset. Then, the Kaplan–Meier survival curves for the OS and RFS of the TCGA datasets were generated according to the optimal cutoff point obtained from X-tile using R package survminer in R version 3.5.2 (https://www.r-project.org/). The R packages rms, survival, and timeROC in R version 3.5.2 (https://www.r-project.org/) were used to evaluate the validity and reliability of the risk score system for OS in TCGA, *RFS* in TCGA and OS in GSE62452 dataset through area under the curve (AUC) of the receiver operating characteristic (ROC) curve analyses. To compare the efficacy of our model with previously reported one, we calculated the AUC of the different models in the same dataset [8]. The higher AUC indicates a better predictability for the model.

### Comparison of the predictive value for the risk score system with other clinical risk factors

To evaluate the predictive power of the risk score system and other potential traditional clinical risk factors, including age (< 60, ≥ 60), sex (male/female), tumor site (head, body and tail), histological grade (G1–G2, G3–G4) and AJCC stage (I–IIa, IIb–IV), we first performed univariable Cox proportional regression and multivariable Cox proportional regression to identified independent predictors. Then we calculated the AUC of each independent predictors to compare the prognostic values of clinical risk factors and our risk score system.

### Functional enrichment analysis

To evaluate the function of the lncRNAs in our risk score system, genes significantly related to the lncRNAs in the risk score system were screened out through calculating the Pearson correlation coefficients between the lncRNAs and the mRNAs in TCGA PC dataset. Then the correlated Genes were enrolled into the enrichment analysis respectively (Pearson correlated coefficient > 0.5 or < − 0.5). ConsensuspathDB (https://cpdb.molgen.mpg.de/) was used to perform GO enrichment analysis and pathway analysis for significantly correlated mRNAs of these lncRNAs in the risk score system. A p-value < 0.01 was regarded as statistically different. Then a dotplot was performed to visualize the significant term of GO enrichment analysis and the significant pathway with the help of the R package ggplot2 in R version 3.5.2 (https://www.r-project.org/).

### Correlation analysis of the risk score system and the infiltration of immune cell in tumor tissues

ESTIMATE was used to assess the infiltration level of immune cells in tumor tissues based on expression data, which was based on single sample gene set enrichment analysis (GSEA). The immune score, which represents the infiltration of immune cells in tumor tissues, was calculated for each PC sample in TCGA PC dataset. The higher immune score indicates a higher level of the infiltration of immune cells in tumor tissues. And correlation analysis of the immune score and the risk score system was conducted to assess the relationship between immune cells infiltration in tumor tissues and the risk score. Besides, we also evaluated the correlation between OS and the immune scores according to the optimal cutoff point obtained from X-tile. Furthermore, using the CIBERSORT method in combination with the LM22 signature matrix, we estimated the differences in the immune infiltration of 22 immune cell types between low- and high-risk PC patients. And we also examined the correlation of the differential infiltrated immune cells with the selected PALncs and the risk score system separately to further evaluate the association between the risk score system and infiltration level of immune cells in tumor tissues.

## Result

### Identification of differentially expressed lncRNAs and prognosis-associated lncRNAs

**Fig. 1:**
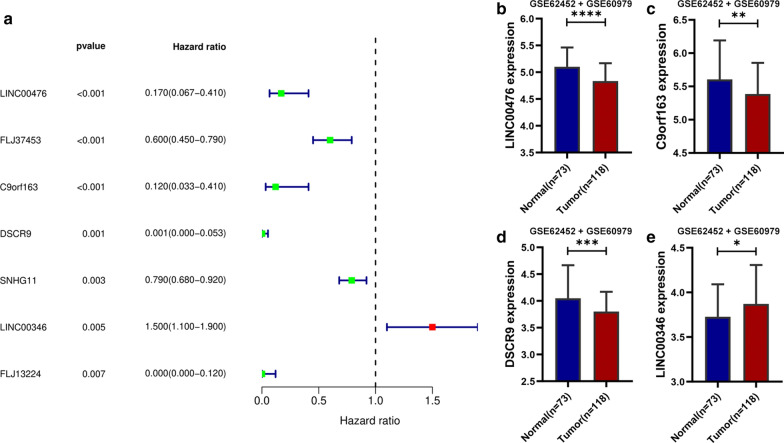
**a** Seven prognosis-associated lncRNAs (PALncs) in TCGA dataset. The univariable Cox proportional regression were used in this analyses. And p < 0.01 was considered significant in this statistical analyses. **b** Differentially expression analysis of Linc00476. **c** Differentially expression analysis of C9orf163. **d** Differentially expression analysis of DSCR9. **e** Differentially expression analysis of LINC00346. LINC00476, c9orf163 and DSCR9 were down-regulated in tumor samples compared to normal pancreas tissue (**b**–**d**). And LINC00346 was up-regulated in tumor samples compared to normal pancreas tissue (**e**)

We screened out 186 lncRNAs from meta-GEO PC dataset through matching the gene symbols of meta-GEO PC dataset with the human genome (Ensemble genome browser 90). Then with the help of the R package limma, we identified 61 DELncs under the thresholds of p-value < 0.05. And the expression value of these 61 DELncs were extracted from the TCGA dataset. Among the 61 DELncs, seven prognosis-associated lncRNAs (PALncs) were screened out through the univariable Cox proportional regression with a p-value < 0.01 (Fig. 1a).

### Establishment and validation of the PALncs-based risk score system

**Fig. 2:**
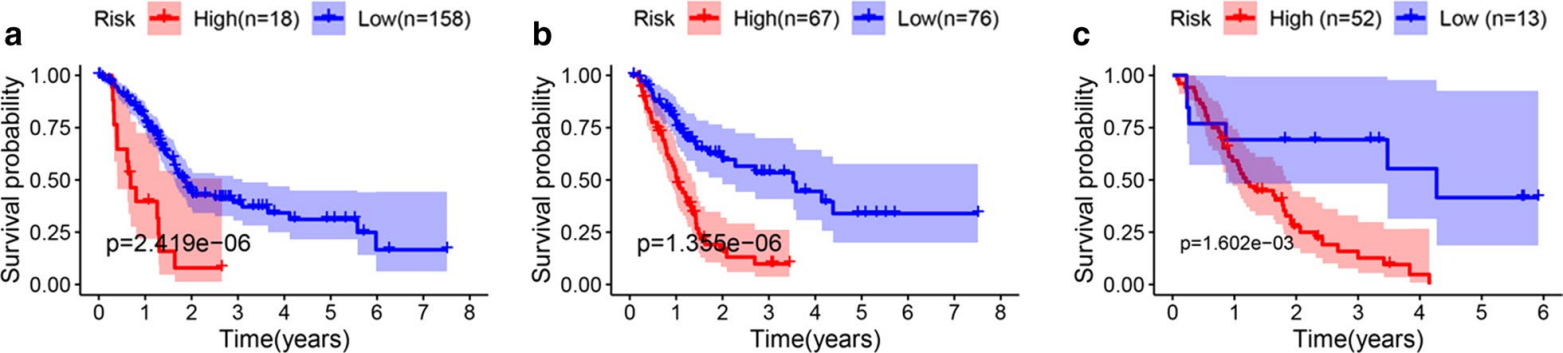
**a** The Kaplan–Meier (KM) survival curves for overall survival (OS) in TCGA dataset in the high- and low-risk groups (p < 0.00001). **b** Kaplan–Meier (KM) survival curves for recurrence-free survival in TCGA dataset in the high- and low-risk group (p < 0.00001). **c** The Kaplan–Meier (KM) survival curves for overall survival (OS) in GSE62452 dataset in the high- and low-risk groups (p < 0.01)

With the help of the R packages survival in R version 3.5.2, multivariate Cox proportional regression analysis was conducted for analyzing the seven PALncs in TCGA dataset to build a prognostic model with the highest discriminated ability for OS. Then a 4-PALncs-based risk score system was developed through the summary of the expression values of these four PALncs multiplied by corresponding coefficients derived from the above multivariable Cox regression analysis: risk score = (− 1.083 * expression value of LINC00476) + (−  1.026 * expression value of C9orf163) + (− 4.120 * expression value of DSCR9) + (0.286 * expression value of LINC00346). The differential expression analysis of these four DELncs were shown in Fig. 1b–e. Then, we classified patients into high- and low-risk groups for OS according to optimal cutoff point (2.055) obtained from X-tile in the TCGA dataset. The Kaplan–Meier survival curve was conducted in the TCGA dataset, which showed significantly different OS and RFS between the high- and low-risk groups (p < 0.00001) (Fig. 2a, b). To further validate these results, the risk score were calculated for 65 patients in GSE62452 dataset. And the KM survival curve was conducted for GSE62452 dataset, which showed significantly different OS between the high- and low-risk groups (p < 0.01) (Fig. 2c).

**Fig. 3:**
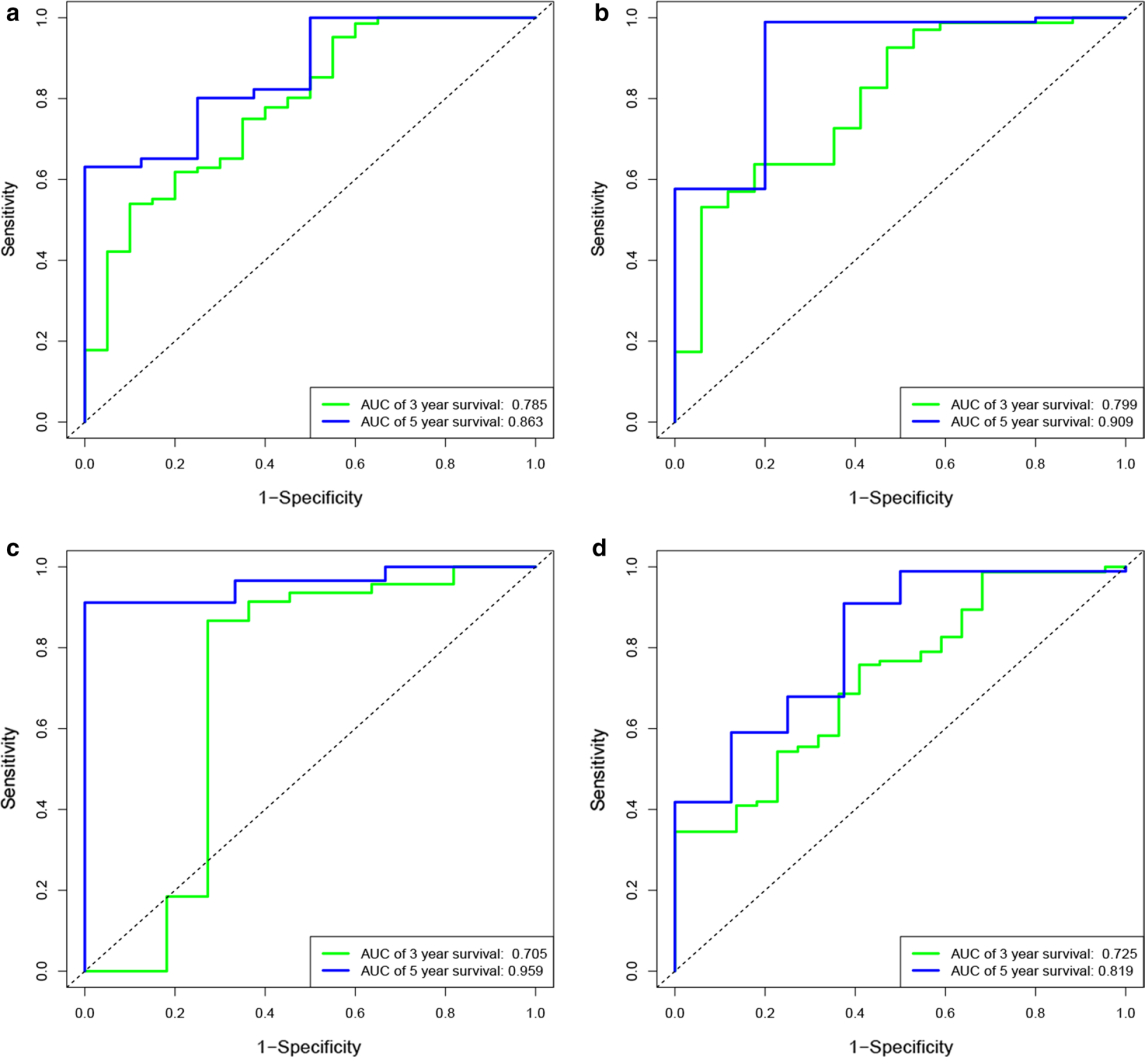
**a** The receiver operating characteristic (ROC) curves for OS in TCGA dataset based on the risk score system. The AUC at the 3- and 5-year survival times was 0.785 and 0.863. **b** The receiver operating characteristic (ROC) curves for RFS in TCGA dataset based on the risk score system. The AUC at the 3- and 5-year survival times was 0.799 and 0.909. **c** The receiver operating characteristic (ROC) curves for OS in GSE62452 dataset based on the risk score system. The AUC at the 3- and 5-year survival times was 0.705 and 0.959. **d** The receiver operating characteristic (ROC) curves for OS based on the previously reported 3-lncRNA-based predictive model. The AUC at the 3- and 5-year survival times was 0.725 and 0.819

The predictive efficacy of the risk score system for OS and RFS in TCGA dataset was assessed through the AUC of the risk score system. The AUC of the risk score for OS in the TCGA datasets was 0.785 at 3 years, 0.863 at 5 years (Fig. 3a). The ROC analysis for RFS in the TCGA dataset was shown in Fig. 3b, which revealed that AUC for RFS was 0.799 at 3 years and 0.909 at 5 years. The AUC for OS in GSE62452 dataset was 0.705 at 3 years, and 0.959 at 5 years (Fig. 3c). Lastly, we compared the efficacy of our model with previously reported one by calculating the AUC of the two models in the same dataset. And we found a higher AUC for our model compared with the reported one (0.785 vs. 0.725 at 3-year OS and 0.863 vs. 0.819 at 5-year OS) (Fig. 3d) [8]. In summary, these results indicated the established risk score system showed satisfactory performance in predicting the OS and RFS for PC.

### Comparison of the predictive value for the risk score system with other clinical risk factors

**Fig. 4:**
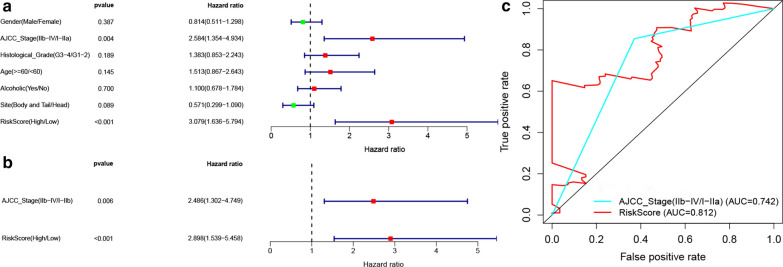
**a**, **b** Univariable and multivariable Cox regression analysis for clinical risk factors in the PC TCGA dataset with OS. **a** Univariable Cox regression analysis. **b** Multivariable Cox regression analysis. **c** ROC curve analyses of the risk score system and AJCC stage, comparing the discriminated ability of the risk score system and AJCC stage through the AUC

The risk score system and other clinical features, including age, sex, tumor site, histological grade and AJCC stage, were evaluated in the TCGA dataset for OS by univariable Cox regression and multivariable Cox regression analysis. Univariable Cox regression analysis revealed that only AJCC stage (I–IIa, IIb–IV) (p = 0.004, HR 2.584, 95% CI 1.354–4.934) and the risk score (high/low) (p < 0.001, HR 3.079, 95% CI 1.636–5.794) were associated with OS (Fig. 4a). And the multivariable Cox regression analysis indicated both risk score system (HR = 2.898, 95% CI, 1.539–5.458, p < 0.001), and AJCC stage (HR = 2.486, 95% CI, 1.302–4.749, p = 0.006) were independent predictors for OS (Fig. 4b). We further compared the discriminated ability of the risk score system and AJCC stage through the AUC, which showed the risk score system had better performance than AJCC stage in predicting OS for PC (Fig. 4c).

### Functional enrichment analysis

The significantly correlated genes of the four PALncs were screened out respectively with the Pearson correlated coefficient > 0.5 or < − 0.5 (p-value < 0.05). Then we performed GO enrichment analysis and pathway analysis separately for the correlated mRNAs of these lncRNAs through ConsensuspathDB (https://cpdb.molgen.mpg.de/). According to the results, we found these four lncRNAs might play an important role in regulating cancer cell behavior and in building the tumor environment. Gene ontology (GO) of LINC00476 and the correlated genes were involved in regulation of glutamate receptor signaling pathway, regulation of cell–matrix adhesion, positive regulation of cell growth, regulation of angiogenesis, regulation of apoptotic signaling pathway, glycolytic process, neutrophil mediated immunity, positive regulation of leukocyte migration, etc. (Additional file 1: Figure S1A). And the pathway analysis showed LINC00476 might play a role in cancer related signaling pathways such as regulation of Ras family activation, glycolysis and gluconeogenesis, senescence and autophagy in cancer, PTK2 signaling, extracellular matrix metabolism, FGF signaling pathway, oxytocin signaling pathway, MAPK signaling pathway, HIF-1 signaling pathway, etc. (Additional file 2: Figure S2A). GO and pathway analysis of C9orf163 indicated it was mainly involved in TNF receptor superfamily (FNFSF) members mediating non-canonical NF-кB pathway, neutrophil degranulation, cytokine signaling in immune system, regulation of chemokine and interleukin production, MET activates PTK2 signaling, ECM-receptor interaction, vasculature development, etc. (Additional file 1: Figure S1B, Additional file 2: S2B). And we found LINC00346 might play a vital role in the carcinogenesis of PC through regulating interleukin signaling, cell cycle checkpoints, cell proliferation, DNA damage stimulus, PLK1 signaling pathway, HIF-1-αtranscription factor network, p53 signaling pathway, etc. (Additional file 1: Figure S1C, Additional file 2: S2C). DSCR9 and its significantly correlated genes might be mainly involved in the tumorigenesis of PC by regulating Ras signaling pathway, p53 signaling pathway, MAPK signaling pathway, and PI3K-Akt signaling pathway, all of which might promote the proliferation and migration of tumor cells (Additional file 1: Figure S1D, Additional file 2: S2D).

### The risk score reflect the infiltration level of immune cell in PC tumor

**Fig. 5:**
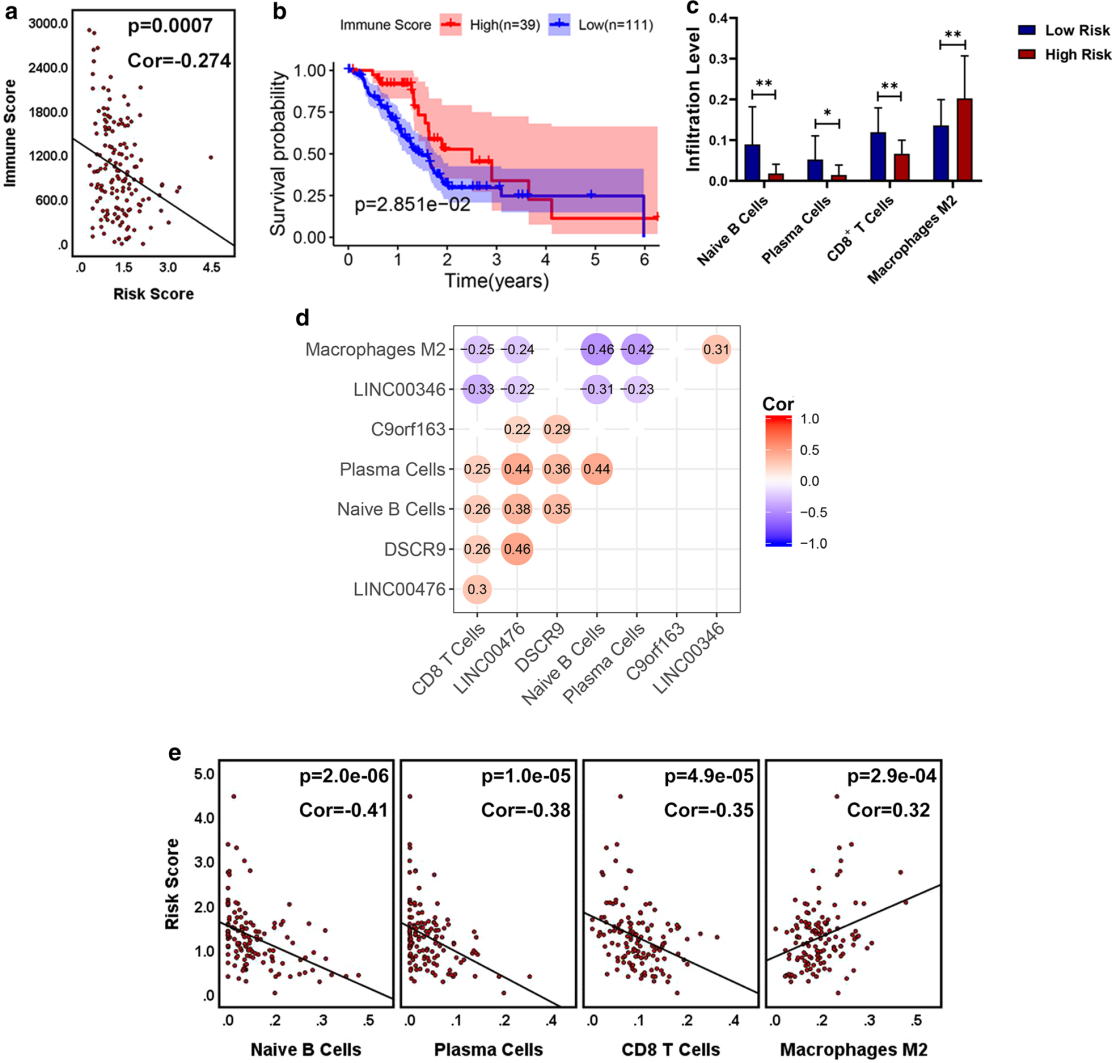
**a** Correlation analysis between the immune score in tumor tissues and the risk score, indicating the immune score was significantly negatively associated with the risk score (Cor = − 0.274, p-value = 0.00070). **b** Kaplan–Meier (KM) survival curves to reveal the correlation between OS and the immune scores. Patients with lower immune score had a shorter OS (p-valve = 0.0285) than those with higher immune score. **c** Bar graphs visualizing significantly different immune cells between high- and low-risk patients. **d** Correlation analysis between the 4 PALncs and the infiltrating level of the significantly different immune cells in tumor tissues. **e** Correlation analysis between the risk score system and the infiltrating level of the significantly different immune cells in tumor tissues

To determine whether the risk score system reflects the infiltration level of immune cell in PC tumor, we calculated the immune score, which represents the infiltration of immune cells in tumor tissues, for each PC sample in TCGA PC dataset. And correlation analysis indicated that the immune score was significantly negatively associated with the risk score in the TCGA PC dataset (Cor = − 0.274, p-value = 0.00070) (Fig. 5a). And the Kaplan–Meier survival curves for OS in TCGA PC dataset revealed that patients with lower immune score had a shorter OS (p-valve = 0.0285) than those with higher immune score (Fig. 5b). Besides, with the help of CIBERSORT method, we assessed the infiltration of 22 immune cell types between patients with low and high risk score (Additional file 3: Figure. S3). We found less infiltration of naive B cells, plasma cells, and CD8+ T cell in PC tissues in patients with high risk score than those with low score (p-value < 0.05) (Fig. 5c). In contrast, more infiltration of macrophages M2 was observed in in patients with high risk score. Correlation analysis of these *four* immune cells and the four PALncs were shown in Fig. 5d. The results revealed that LINC00476, LINC00346 and DSCR9 were all highly associated with immune infiltration in PC. Besides, correlation analysis of these *four* immune cells and the risk score system further revealed that the infiltration level of B cell naïve, plasma cells, and CD8+ T cells are negatively correlated to the risk score system, while macrophages M2 positively correlated to the risk score system (Fig. 5e). Taken together, these results suggest the risk score system might negatively reflects the infiltration level of immune cell in PC tumor, especially for CD8+ T cell, B cell naïve, and plasma cells, which are responsible for adaptive anti-tumor immunity.

## Discussion

In this study, we established a 4-LncRNAs based risk score system to predict prognosis for PC, which showed a reliable performance. KM survival analysis indicated significantly different survival between patients with higher risk score and lower score. The AUC of ROC curve analysis of this model for 3- and 5-year OS in the TCGA dataset were 0.785 and 0.863, respectively. In addition, the model is validated in the GEO dataset, which also presented satisfactory AUC of 0.705 and 0.959 for 3- and 5-year OS. In previous study, Wu et al. [8] introduced a three-lncRNAs (MIR600HG, AL137789.1, and AC079015.1) based prognostic signature for PC. Of note, we compared our model with the prognostic signature by Wu et al. in the TCGA PC dataset. And we found a higher AUC for our model compared with the model by Wu et al. (0.785 vs. 0.725 at 3-year OS and 0.863 vs. 0.819 at 5-year OS), which suggest a superior predictive value of our model. Taken these together, the four-PALncs based risk score system in our study presents strong predictive power for PC, which may facilitate clinicians in identifying more aggressive tumors and making more appropriate therapeutic decision individually. And we recommend close follow up for patients in the high-risk group due to a high risk of tumor recurrence.

Among the four LncRNAs identified in the study, LINC00476, c9orf163 and DSCR9 were down-regulated in tumor samples compared to normal pancreas tissue. Besides, the expression of LINC00476, c9orf163 and DSCR9 was *positively* correlated with PC patients’ survival. These suggested LINC00476, c9orf163 and DSCR9 might act as tumor suppressors in PC. Wang et al. [9] found LINC00476 was a protective prognostic probes in anaplastic glioma by performing cox regression in GSE16011 dataset. Similar results was presented in prostate cancer [10]. However, the molecular mechanism by which LINC00476 regulated cancer cell’s biological function or behavior is largely unknown. Studies found LINC00476 might be involved in phosphatidylinositol signaling system, Ras signaling pathway, Rap1 signaling pathway, and TNF signaling pathway. Experiments are needed to explore the mechanism for LINC00476 in PC in future study.

There are less studies for C9orf163 and DSCR9 in the context of cancer. In consistent with our results, Wang et al. [11] reported c9orf163 was deleted in Head and neck squamous cell carcinoma. AS for DSCR9, Zhu et al. [12] found that the hypermethylated of 3′-gene downstream region of DSCR9 might silence the expression of DSCR9, and it could be a biomarker capable of distinguishing prostate malignancy from normal prostate tissues. These data partly support our finding, in which C9orf163 and DSCR9 might be a tumor suppressor (Additional file 1: Figure S1D, Additional file 2: S2D). In the molecular networks, C9orf163 was shown to be connected with TNF receptor superfamily (FNFSF) members mediating non-canonical NF-кB pathway, neutrophil degranulation, cytokine signaling in immune system, regulation of chemokine and interleukin production pathway, etc. (Additional file 1: Figure S1B, Additional file 2: S2B). These suggest that C9orf163 might contribute to building tumor microenvironment via affecting cytokine and chemokine related signaling. More studies are needed to clarify the target genes and detailed molecular mechanism of c9orf163 and DSCR9.

Unlike the above three LncRNAs, LINC00346 is up-regulated in tumor samples compared to normal pancreas tissue and higher expression of LINC00346 is associated with worse survival. These indicate LINC00346 might be an oncogene in PC. Indeed, Peng et al. [13] found knockout (KO) of LINC00346 impairs pancreatic cancer cell proliferation, tumorigenesis, migration, and invasion ability. And mechanistically, LINC00346 promotes PC progression by interacting with CTCF, leading to c-Myc activation. Other studies also addressed the oncogenic role of LINC00346 in bladder cancer and gastric cancer. Taken these together, LINC00346 is a critical oncogene in PC tumorigenesis and is also an effective biomarker for prediction for patients’ prognosis.

The pathway enrichment for these four lncRNAs revealed that all of them might play an important role in the chronic and smoldering inflammation in the tumor microenvironment (TME) of PC, perhaps leading to the hypoxia or hyaluronan-induced development of desmoplastic stoma, deactivation of anti-tumor immune cells (e.g. CD8+ T cells, CD4+ T cell, and NK cells), recruitment of anti-inflammatory immune cells (e.g. macrophages M2 and neutrophils) (Additional file 2: Figure. S2), which in somehow were reflected through correlation analysis of these four immune cells and these four PALncs (Fig. 5d). Tumor-infiltrating lymphocytes (TIL) in tumor stroma are classically considered to be crucial for the elimination of malignant cells and prevention of tumor progression [14]. The complex aggregates of cytotoxic lymphocytes, B lymphocytes (e.g. plasma cells) and dendritic cells are considered as tertiary lymphoid structures (TLS) [15]. And the presence of TLS in tumor tissue has largely associated with a favorable prognosis for patients with solid tumors [16]. Recent studies have revealed that cytotoxic CD8+ T cells are important effector cells in adaptive immunity, specifically recognizing and clearing tumor cells and increasing number of CD8+ T cells was associated with prolonged survival in cancer [17]. Previous studies found that the infiltration of CD20+ B cells in ovarian cancer, non-small lung carcinoma and cervical cancer was correlated with improved survival and lower relapse rates [17, 18]. And TLS were proved to contain large aggregates of plasma cells which were positively associated with antitumor response. But the role played by infiltrating B cells is still poorly understood. In this study, we found the risk score system negatively reflected the infiltration level of anti-tumor immune cells, especially for CD8+ T cell, B cell naïve, and plasma cells, indicating the presence of higher density of TLS in tumor tissues in patients with lower risk score (Fig. 5c, e). Besides, high risk score might indicate high infiltration level of macrophages M2, which play a critical role in tumor progression (Fig. 5c, e). It is reported that macrophages M2 might inhibit inflammation through secreting cytokines such as IL-10 and TGF-β. And macrophages M2 could inhibit T cell proliferation and differentiation, and promote proliferation of tumor cells and angiogenesis of tumor [19]. Taken together, we supposed that the transformation of pro-inflammatory to anti-inflammatory in the TME was much more thorough in PC patients with higher risk score than those with low risk score, which increases the tumor progression and angiogenesis.

## Conclusion

In our current study, we established a four prognosis-associated lncRNAs (PALncs) based risk score system, whose power of prediction was better that that of conventional AJCC-stage. And we also found the risk score system negatively reflected the infiltration level of anti-tumor immune cells (e.g. CD8+ T cells, B cell naïve, and plasma cells) and positively correlated to the infiltration level of macrophages M2, indicating the presence of higher density of TLS in tumor tissues in patients with lower risk score in PC, and more thorough transformation of pro-inflammatory to anti-inflammatory in the TME of PC patients with higher risk score. Future studies are needed to further validate our model and to explore the molecular function and mechanism of these PALncs in regulating anti-tumor immunity.

## Supplementary information

**Additional file 1: Figure S1.** GO enrichment analysis for the correlated mRNAs of these 4 lncRNAs. (A) LINC00476. (B) C9orf163. (C) LINC00346. (D) DSCR9.

**Additional file 2: Figure S2.** Pathway enrichment analysis for the correlated mRNAs of these 4 lncRNAs. (A) LINC00476. (B) C9orf163. (C) LINC00346. (D) DSCR9.

**Additional file 3: Figure S3.** The differences of 22 immune cells between the low-risk and high-risk groups.

## Data Availability

The datasets generated and analyzed during the current study are available in The Cancer Genome Atlas (TCGA), https://cancergenome.nih.gov/, and Gene Expression Omnibus, https://www.ncbi.nlm.nih.gov/geo/.

## Abbreviations

PC: Pancreatic cancer
TCGA: The Cancer Genome Atlas
GEO: Gene Expression Omnibus
PALncs: Prognosis-associated lncRNAs
OS: Overall survival
RFS: Recurrence-free survival
KM: Kaplan–Meier
DELnc: Differentially expressed lncRNAs
AUC: Area under the curve
ROC: Receiver operating characteristic
GO: Gene oncology
GSEA: Gene set enrichment analysis
HR: Hazard ratio
CI: Confidence interval
AJCC: The American Joint Committee on Cancer
KEGG: Kyoto Encyclopedia of Gens and Genomes
MET: Epithelia–mesenchymal transition
TIL: Tumor-infiltrating lymphocytes
TLS: Tertiary lymphoid structures
TME: Tumor microenvironment
