# Supplementary figures and images for: A four prognosis-associated lncRNAs (PALnc) based risk score system reflects immune cell infiltration and predicts patient survival in pancreatic cancer
Source: Cancer Cell Int. 2020 Oct 9;20:493. doi: 10.1186/s12935-020-01588-y (PMC7547431; doi:10.1186/s12935-020-01588-y)

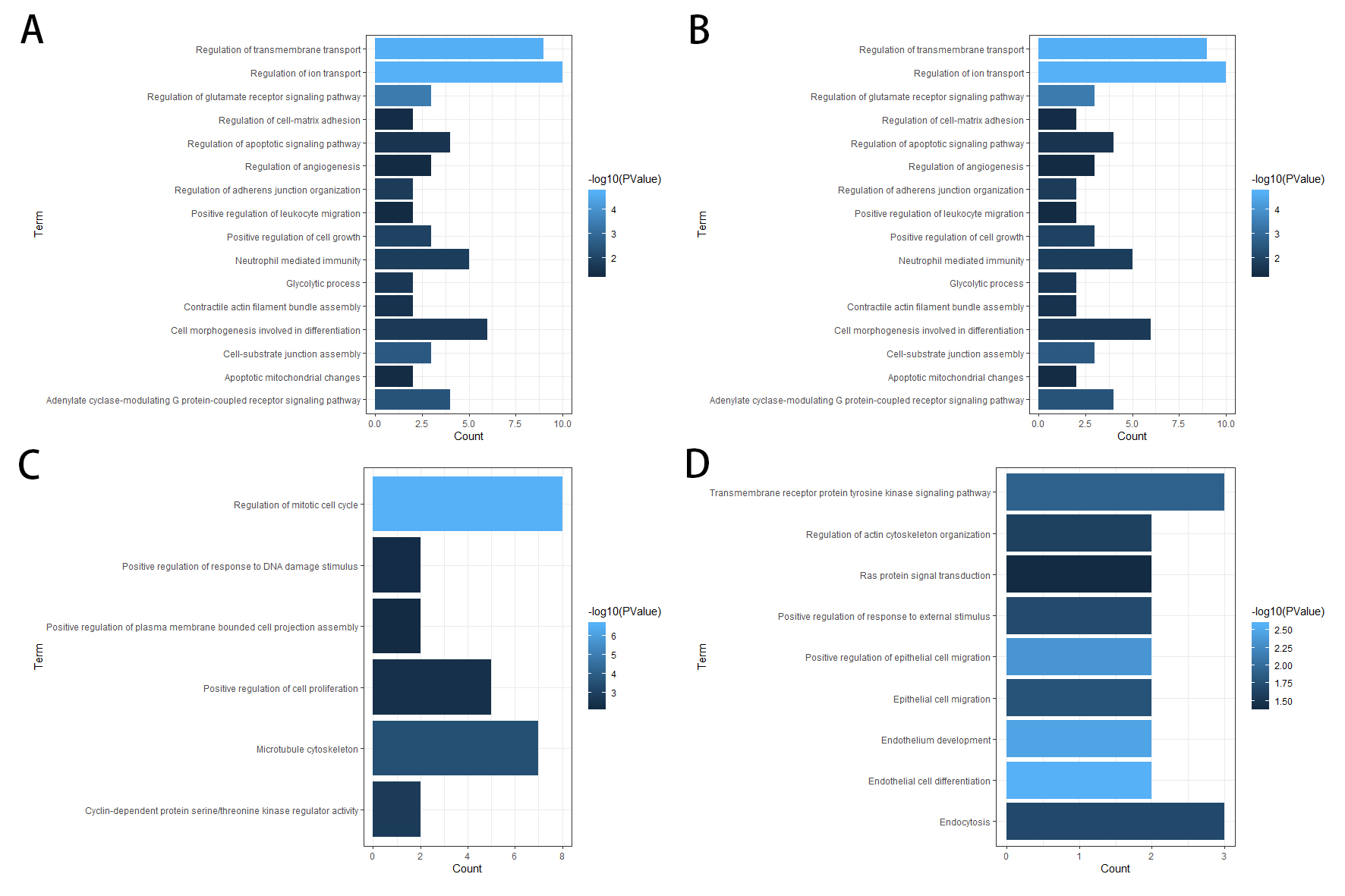

Supplement: Supplementary file 1 — Additional file 1: Figure S1. GO enrichment analysis for the correlated mRNAs of these 4 lncRNAs. (A) LINC00476. (B) C9orf163. (C) LINC00346. (D) DSCR9. [file 12935_2020_1588_MOESM1_ESM.jpg]

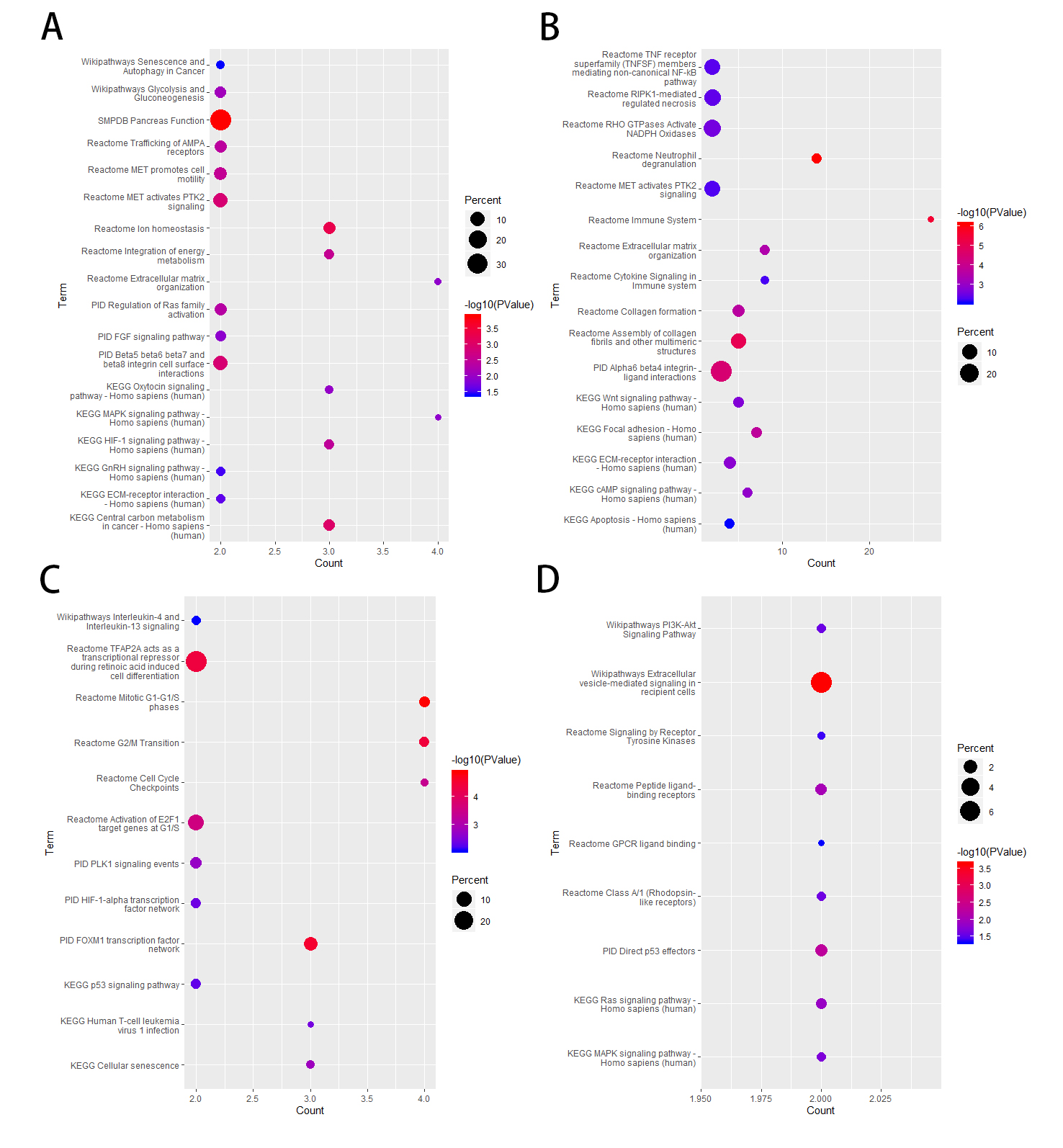

Supplement: Supplementary file 2 — Additional file 2: Figure S2. Pathway enrichment analysis for the correlated mRNAs of these 4 lncRNAs. (A) LINC00476. (B) C9orf163. (C) LINC00346. (D) DSCR9. [file 12935_2020_1588_MOESM2_ESM.jpg]

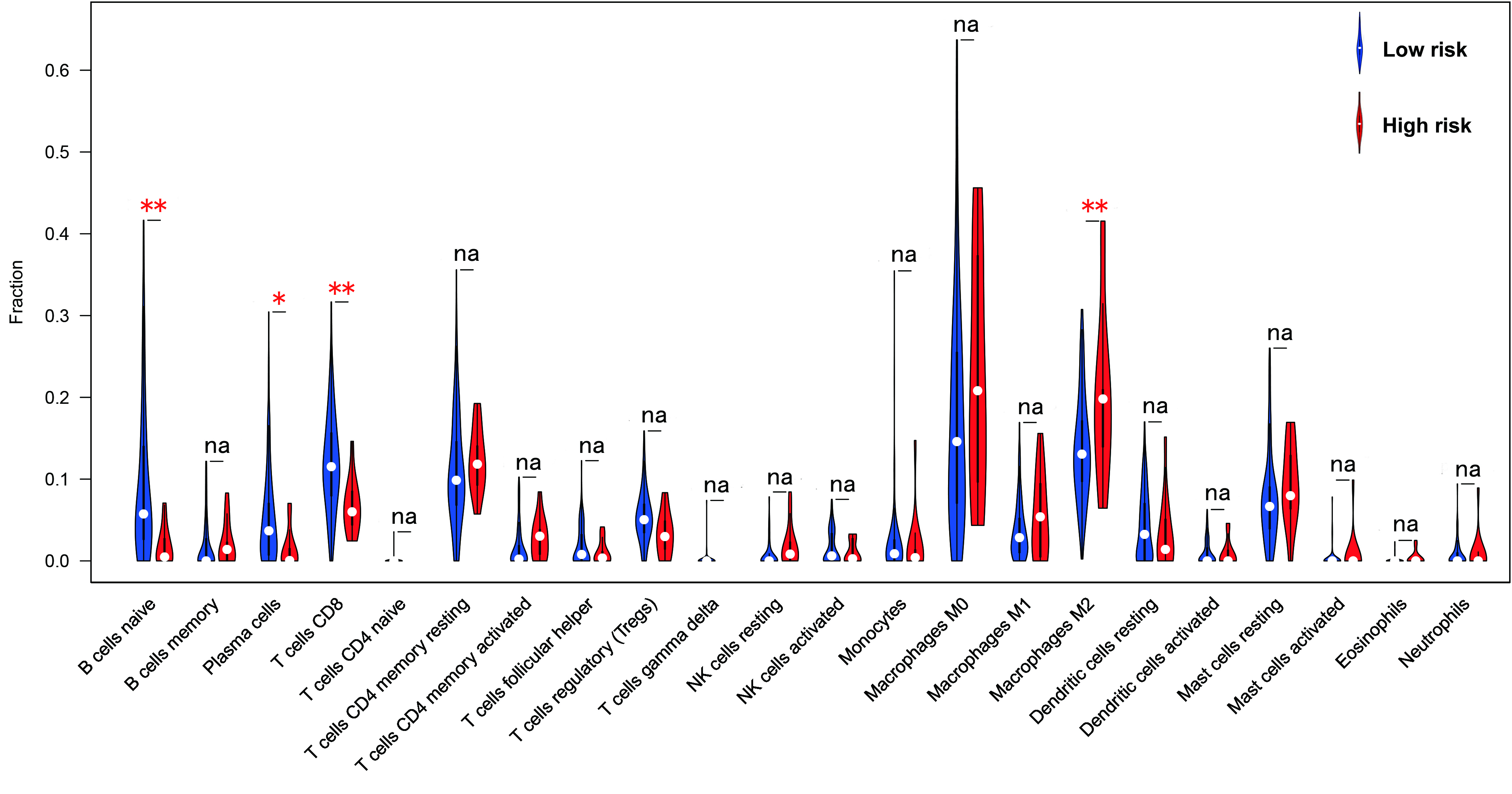

Supplement: Supplementary file 3 — Additional file 3: Figure S3. The differences of 22 immune cells between the low-risk and high-risk groups. [file 12935_2020_1588_MOESM3_ESM.jpg]
